# Adventitial Cystic Disease in the Popliteal Artery Diagnosed by Intravascular Ultrasound Imaging

**DOI:** 10.7759/cureus.34362

**Published:** 2023-01-29

**Authors:** Eiji Miyauchi, Hideki Okui, Toshinori Yuasa, Naoya Oketani, Mitsuru Ohishi

**Affiliations:** 1 Division of Cardiology, Kagoshima City Hospital, Kagoshima, JPN; 2 Division of Cardiology, Kanoya Medical Center, Kanoya, JPN; 3 Department of Cardiovascular Medicine and Hypertension, Graduate School of Medical and Dental Sciences, Kagoshima, JPN

**Keywords:** intravascular ultrasound (ivus), popliteal artery, intermittent claudication, pad(peripheral artery disease), cystic adventitial disease

## Abstract

The prevalence of peripheral artery disease (PAD) has been increasing in parallel with the increasing prevalence of the atherosclerotic disease. Therefore, we have to be familiar with the diagnostic approach used for ischemic symptoms in the lower limbs. Adventitial cystic disease (ACD) is rare but not negligible as one of the differential diagnoses of intermittent claudication (IC). Although duplex ultrasound and magnetic resonance imaging (MRI) are helpful tools for the diagnosis of ACD, further imaging modality is needed to avoid misdiagnosis. A 64-year-old man with a mitral valve prosthesis presented to our hospital with a one-month history of IC in the right calf after walking for approximately 50 meters. On physical examination, the pulse in the right popliteal artery was not palpable, nor were the dorsal pedis artery and posterior tibial artery, although there were no other symptoms of ischemia. His right ankle-brachial index (ABI) was 1.12 at rest but decreased to 0.50 after exercise. Three-dimensional computed tomography (CT) angiography revealed a severe stenotic lesion approximately 70 mm long in the right popliteal artery. Therefore, we diagnosed PAD in the right lower limb and planned endovascular therapy. The stenotic lesion was markedly reduced on catheter angiography when compared with CT angiography. However, intravascular ultrasound (IVUS) detected little atherosclerosis and cystic lesions within the wall in the right popliteal artery that did not involve the arterial lumen. Especially, IVUS clearly demonstrated that the crescent-shaped cyst compressed the arterial lumen eccentrically and other cysts surrounded the lumen circumferentially like petals. Because IVUS revealed these cysts to be extravascular structures, the patient was subsequently thought to have ACD of the right popliteal artery. Fortunately, his cysts reduced in size spontaneously and his symptoms disappeared. We have monitored the patient’s symptoms, ABI, and findings on duplex ultrasound for seven years, during which there has been no recurrence. In this case, we diagnosed ACD in the popliteal artery by IVUS rather than duplex ultrasound and MRI.

## Introduction

The prevalence of peripheral artery disease (PAD) has been increasing in parallel with the increasing prevalence of the atherosclerotic disease [1]. Therefore, we have to be familiar with the diagnostic approach used for ischemic symptoms in the lower limbs. Adventitial cystic disease (ACD) is rare but not negligible as one of the differential diagnoses of intermittent claudication (IC) [2-4]. A non-atherosclerotic vascular disease, ACD is caused by the formation of mucinous material within the adventitia of the affected vessel. An adventitial mucoid cyst, which compresses the arterial wall, subsequently causes narrowing of the vessel lumen and limb ischemia, resulting in IC.

Although duplex ultrasound, computed tomography angiography (CTA), magnetic resonance angiography, and magnetic resonance imaging (MRI) are undoubtedly helpful tools for the diagnosis of ACD, our experience in this case suggests that intravascular ultrasound (IVUS) may also be a useful imaging tool.

## Case presentation

The case was a 64-year-old Asian male who presented to our hospital with a one-month history of IC in the right calf after walking approximately 50 meters. He had received a heart valve prosthesis three years earlier for infectious endocarditis. There was no history of trauma, manual labor, or risk factors for cardiovascular disease. On physical examination, the right popliteal, dorsal pedis, or posterior tibial artery was not palpable, although there were no other symptoms of ischemia. His ankle-brachial index (ABI) was 1.12 at rest but decreased to 0.50 on the right after exercise. An electrocardiogram and chest radiograph were unremarkable. An echocardiogram revealed that there were no problems with the mitral prosthetic valve.

**Figure 1:**
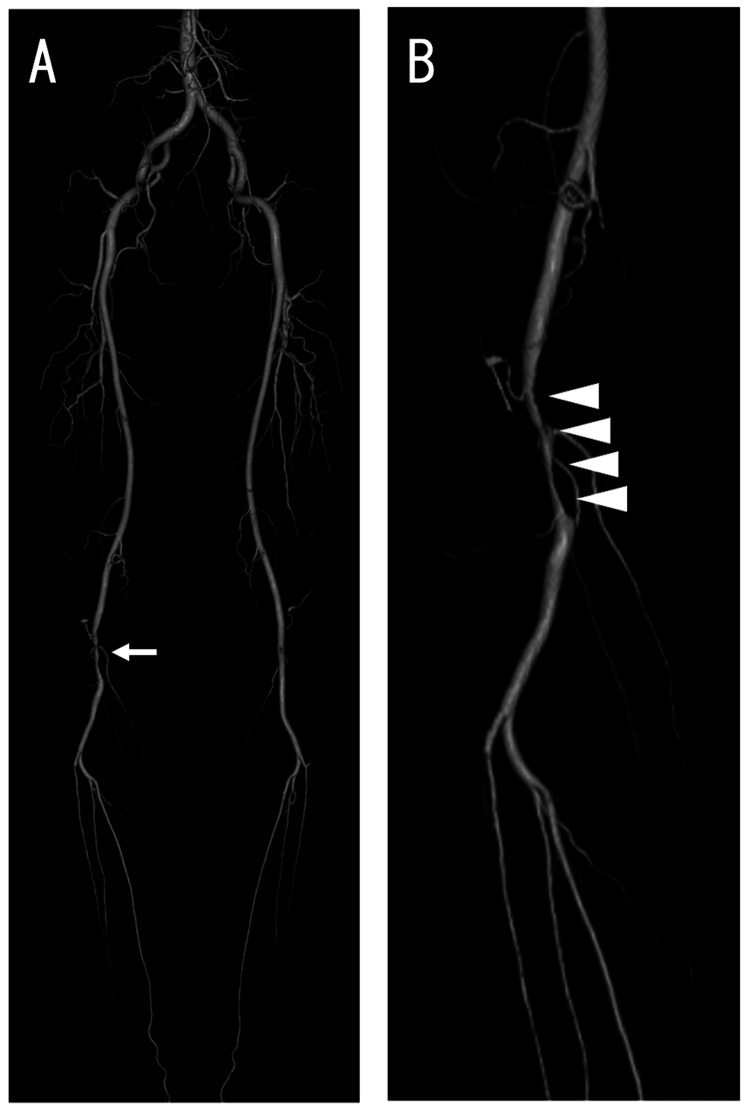
Three-dimensional computed tomography angiography of the lower limb. (A) Frontal view. Approximately 70 mm long severe stenotic lesion (white arrow) was detected in the right popliteal artery. No other specific stenosis was found. (B) Left anterior oblique view. The lesion (white arrowhead) was specifically localized in the right popliteal artery and looked like hourglass.

Three-dimensional CTA revealed a severe stenotic lesion measuring approximately 70 mm in length in the right popliteal artery (Figure 1).

**Figure 2:**
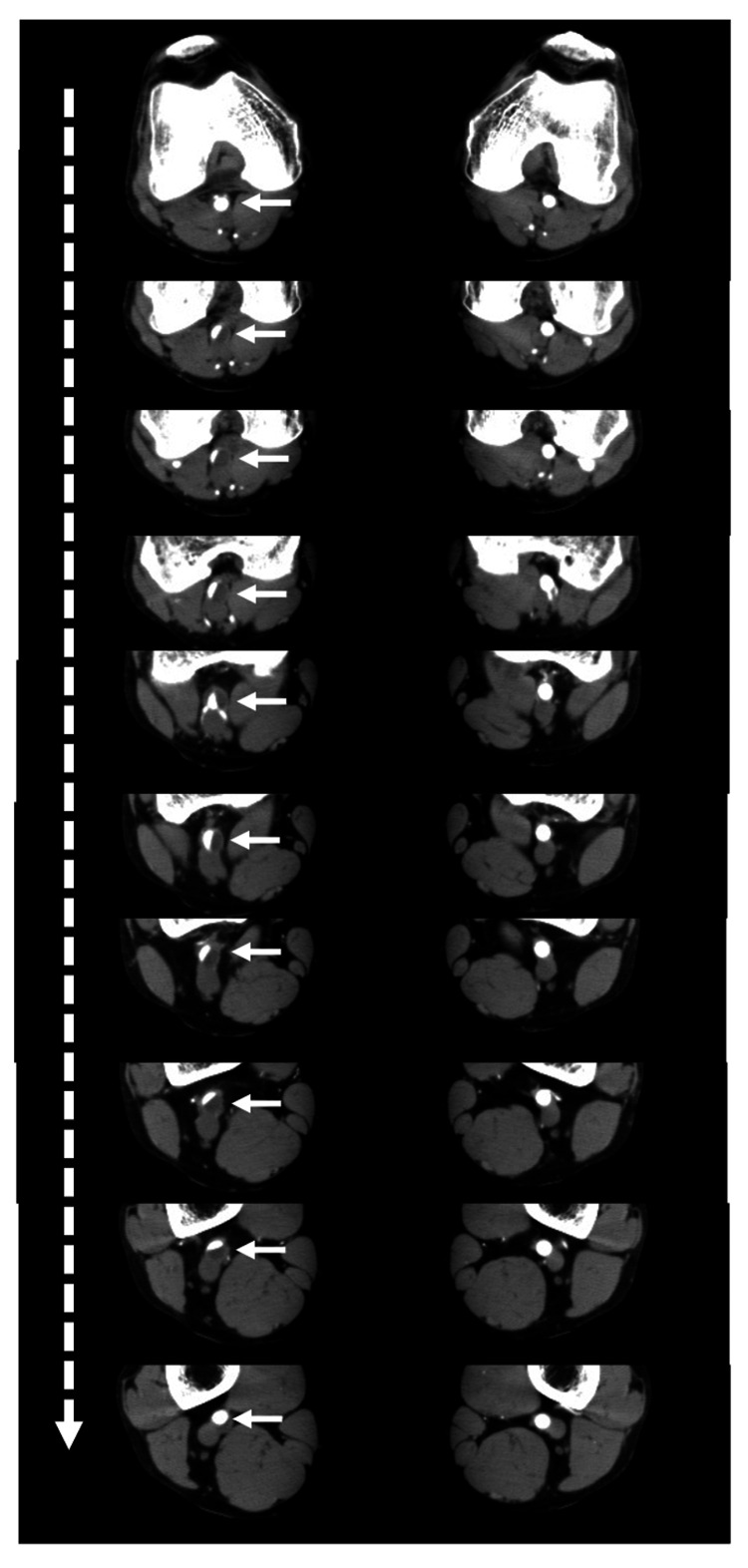
Cross-section of the computed tomography around the knee joint. In the continuous left panels, stenotic lesion (white arrow) was detected in the right popliteal artery; on the other hand, in the right panels, there were no stenotic lesions in the left popliteal artery. The white dotted arrows indicate the direction from proximal to distal.

The lesion had an hourglass-like shape. A cross-sectional CT image also showed a severe stenotic lesion in the right popliteal artery (Figure 2).

**Figure 3:**
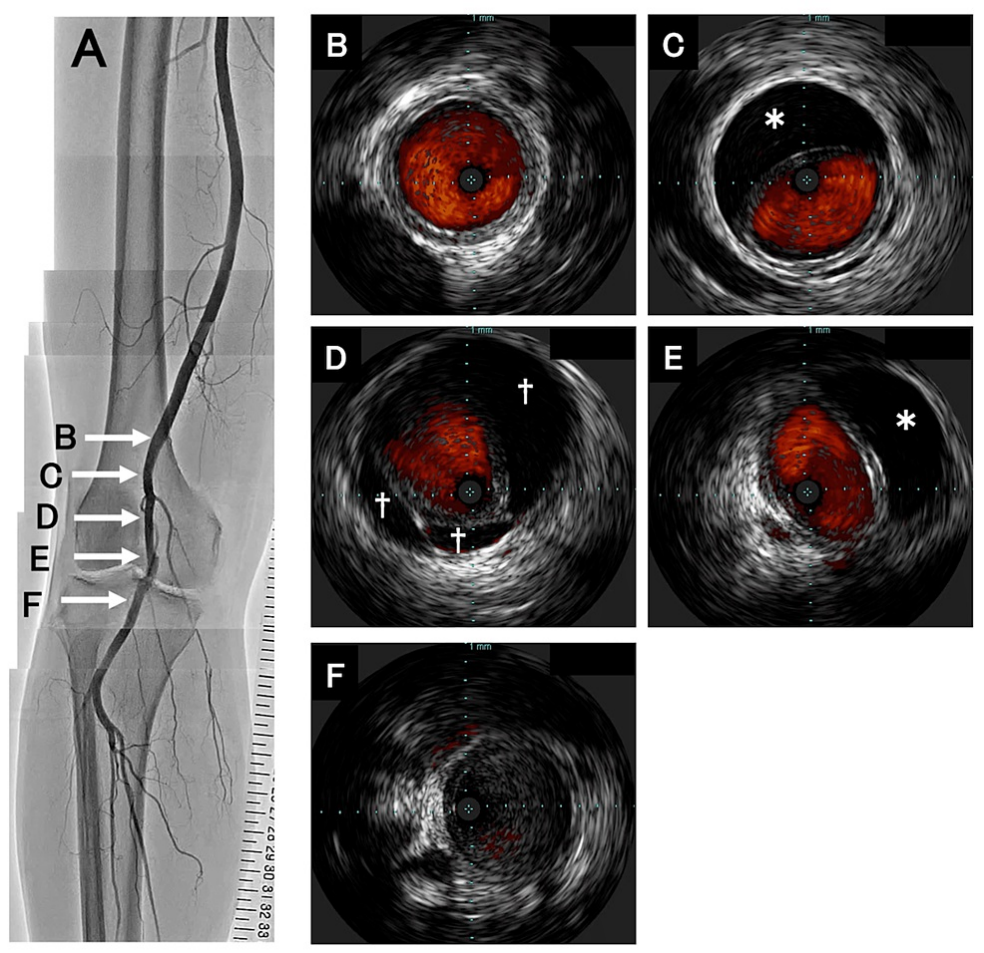
Images on the right lower limb obtained by catheter angiography and intravascular ultrasound images. (A) Catheter angiography of the right lower limb. This image was made by merging several images into one. Stenotic lesion was angiographically reduced in the middle segment of the right popliteal artery. (B-F) Intravascular ultrasound cross-sectional images obtained from a 20 MHz device from proximal to distal popliteal artery. (B) Proximal popliteal artery shows typical three-layer appearance of arterial wall. (C), (E) Eccentric compression of the arterial lumen by a crescent-shaped cyst (asterisk). (D) Cysts (dagger) surround lumen circumferentially like a petal. (F) Distal popliteal artery shows typical three-layer appearance of arterial wall.

Therefore, we diagnosed PAD in the right leg and planned invasive catheter angiography with a plan for possible intervention. However, contrary to our expectations, catheter angiography revealed only a minor stenotic lesion in the right popliteal artery (Figure 3A).

**Figure 4:**
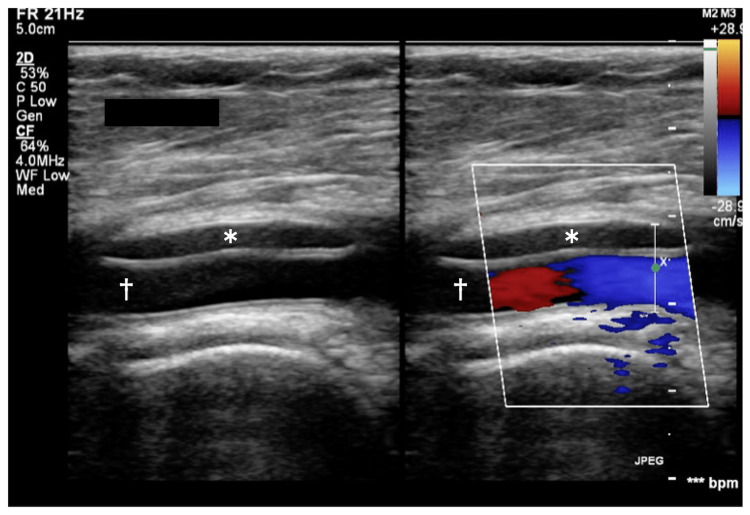
Duplex ultrasound of right popliteal artery. Echo-free space (asterisk) was found in the right popliteal artery (dagger). The left panel is the B mode image, and right panel is the color flow Doppler image.

Compared with the findings on CTA, the stenotic lesion seemed to be markedly reduced. Therefore, the origin of the stenotic lesion was suspected to be thromboembolism. IVUS showed little in terms of atherosclerosis or thrombus but revealed cystic lesions in the arterial wall of the right popliteal artery (Figure 3B-3F). Especially, IVUS clearly demonstrated that the crescent-shaped cysts compressed the arterial lumen eccentrically and other cysts surrounded the lumen circumferentially like petals in the affected popliteal artery. Because IVUS revealed these cysts to be extravascular structures, the patient was subsequently thought to have ACD of the right popliteal artery. After the angiography, we also performed a duplex ultrasound and MRI. Duplex ultrasound showed that the true lumen was compressed by echo-free spaces (Figure 4).

**Figure 5:**
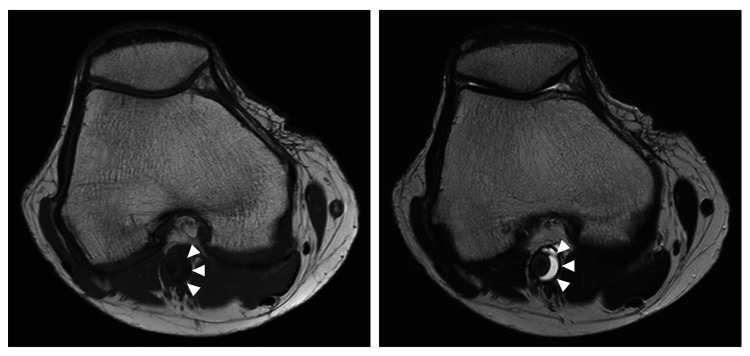
Magnetic resonance images of the right popliteal artery. These cystic lesions (white arrowhead) exhibited low signal intensity on T1-weighted image (left panel) and high signal intensity on T2-weighted image (right panel).

On MRI, the cystic lesions showed low signal intensity on a T1-weighted image and high signal intensity on a T2-weighted image (Figure 5).

These findings were consistent with ACD. Fortunately, the cysts decreased in size spontaneously, and his symptoms disappeared. We followed the patient annually using ABI and duplex ultrasound of the lower limb discussing with our vascular surgeons, and there was no recurrence for seven years since his discharge.

## Discussion

Atkins and Key reported the first case of ACD in 1947 [5]. Since then, more than 500 case reports and case series have been published. The majority of the ACDs were in the popliteal artery: It has been reported that approximately 85% of ACDs occur in the popliteal artery, and the others in the iliac, femoral, and radial artery and femoral and popliteal vein [6]. The rate of occurrence was about one in 1200 cases of claudication [7]. As far as gender is concerned, ACD was predominant in males, and the odds ratio was 15 compared to females [8]. The etiology of ACD was not clearly elucidated. Histologically, ACD cysts were composed of proteoglycans, mucoproteins, and mucopolysaccharides, and they include a high content of hyaluronic acid similar to ganglia. However, the triggers of the ACD cyst formation were not clear. Four possible theories of cyst formation were advocated [9]: traumatic, degenerative, developmental, and synovial. In traumatic theory, the cysts are formed due to repetitive micro-trauma by stretching and distorting of an artery adjacent to a joint. In degenerative theory, the cysts formations are associated with systemic connective tissue disease. In development theory, undifferentiated mesenchymal cells may migrate from the adjacent joints into the adventitia of blood vessels during the embryonic period. In synovial theory, this is recognized as the main reason for ACD now, that is, capsular synovial structures are growing and tracking in the adventitia along the vascular branches. It is supported by the fact that adventitial cysts occur mainly in large arteries or veins which overlie a joint, and the morphology of the cysts is similar to that of ganglia. Since Shute et al. first reported [10] a direct communication between the knee joint and an adventitial cyst in 1973, many authors have reported similar findings, although the connection with the joint could not be identified on imaging in our case.

In the treatment for ACD, Koppensteiner et al. reported [11] that endovascular therapy (EVT) had a high recurrence rate, and percutaneous cyst aspiration was not effective due to the high viscosity of the gelatinous material. Then surgical revascularization was the first-line therapy for patients with ACD [12]. The initial success rate was higher, and the recurrence rate was lower compared to those of EVT. However, it was reported that the ACD cyst disappeared spontaneously [13]. The possible mechanisms were cyst rupture or drainage into the joint space, and they were supported by the arthroscopic, intraoperative, or magnetic resonance angiography findings of direct communication between the popliteal adventitial cyst and the knee joint. But the cysts that spontaneously disappeared could recur; therefore, the exact follow-up is mandatory in such cases. Fortunately, there was no recurrence in our case for seven years.

ACD is rare and may be misdiagnosed as PAD. Once misdiagnosed as PAD and treated with EVT, stenotic lesions may recur [11,14,15]. Therefore, ACD is not negligible in the differential diagnosis of IC [4]. To investigate the etiology of the stenotic lesion, we have to be familiar with the imaging examinations, such as duplex ultrasound and magnetic resonance angiography. The etiology of ACD is somewhat different from that of PAD. ACD is a non-atherosclerotic vascular disease in which mucinous material forms within the adventitia of the affected vessel. The adventitial mucoid cyst, which compresses the arterial wall, subsequently causes the narrowing of the lumen and induces IC or limb ischemia. Given that mucoid cysts, the main cause of ACD, are located in the adventitia of the affected vessel, accurate diagnosis requires the selection of imaging modalities that can accurately estimate not only the vessel lumen but also the vessel walls and external components. In our case, findings on CT and catheter angiography indicated that the arterial wall might be compressed from the outside. The luminal stenosis would have an hourglass-like appearance if the cysts are concentric and a scimitar-like appearance if eccentric. MRI is better than CT or catheter angiography for the diagnosis of ACD because it depicts the wall of the vessels and the surrounding anatomical structures, including the vessel lumen. MRI can exclude any other pathology as a differential diagnosis, such as PAD, popliteal aneurysm, popliteal artery entrapment syndrome, popliteal ganglion, or soft tissue tumor. Duplex ultrasound is a suitable diagnostic modality because of its non-invasiveness, effectiveness, and general availability, and it is also useful for follow-up. Duplex ultrasound followed by MRI appears to be the better imaging strategy for ACD.

IVUS is also a useful imaging modality [16]. First, it has a higher frequency than transcutaneous ultrasound (20 MHz versus 10 MHz) and allows a more detailed evaluation of the morphology of the three layers of the vessel wall (i.e., the intima, media, and adventitia) because of its higher spatial resolution. In ACD, in which mucinous material collects within the adventitia of the affected vessel, IVUS shows multiloculated echo-free spaces that have a petal-like or crescent-like appearance and exist circumferentially between the media and adventitia, with little plaque and a normal intima. Second, IVUS can evaluate not only vessel wall structures but also extravascular structures to some extent. IVUS can easily differentiate between ACD, thrombosis, PAD, and dissection, which are abnormalities of the lumen or vessel wall, as well as popliteal ganglion, Baker's cyst, and popliteal artery compression syndrome, which are extravascular abnormalities. Third, because IVUS is currently the standard device used in EVT, interventionists are familiar with the analysis of the findings using this imaging modality. If ACD is misdiagnosed as PAD before an endovascular procedure, interventionalists can still identify ACD during the procedure if IVUS is performed and shows no atherosclerosis in the affected vessel and an echo-free space within the adventitia. In our case, the stenosis in the popliteal artery appeared to be milder on angiography than on CT, which raised suspicion for thrombosis. However, IVUS showed multiloculated echo-free spaces around the stenotic artery. The intimal and medial layers of the artery were normal, and the lesions were derived from the outer layer. The arterial wall was normal in the proximal and distal portions, and there was no evidence of arteriosclerosis. These findings suggest that IVUS is a reliable tool for the identification of ACD. Although invasive, it can be a diagnostic alternative to duplex ultrasound or MRI in patients undergoing angiography.

## Conclusions

ACD is rare but not negligible as one of the differential diagnoses of IC. Although duplex ultrasound and MRI are undoubtedly helpful as diagnostic tools for ACD, our experience in this case suggests that IVUS may also be a useful imaging modality. We should be aware of the pathophysiological implications of ACD, which can be diagnosed more reliably with IVUS in addition to duplex ultrasound and MRI.
